# Statement of retraction: [thiophen urea derivatives as a new class of hepatitis C virus entry inhibitors]

**DOI:** 10.1080/14756366.2021.1927378

**Published:** 2021-06-24

We, the Editors and Publishers of the Journal Enzyme Inhibition and Medicinal Chemistry, have retracted the following article:

Thiophen urea derivatives as a new class of hepatitis C virus entry inhibitors; Hyung Chul Ryu, Marc Windisch, Jee Woong Lim, Inhee Choi, Eun Kyu Lee, Hye Hyun Yoo, Tae Kon Kim; Journal of Enzyme Inhibition and Medicinal Chemistry; Volume 36, Issue 1, Pages 462–468 (2021); 10.1080/14756366.2020.1870456

This article is retracted because the authors did not have permission from IPK to publish the data. In addition, concerns were raised regarding the integrity of the data in the article. The second author was also added to the authorship list without his consent. The authors have agreed that due to the above concerns, the article should be retracted.

We have been informed in our decision-making by our policy on publishing ethics and integrity and the COPE guidelines on retractions. The retracted article will remain online to maintain the scholarly record, but it will be digitally watermarked on each page as “Retracted”.

